# Leveraging the potential of 1.0-mm i.d. columns in UHPLC-HRMS-based untargeted metabolomics

**DOI:** 10.1007/s00216-024-05588-z

**Published:** 2024-10-24

**Authors:** Danila La Gioia, Emanuela Salviati, Manuela Giovanna Basilicata, Claudia Felici, Oronza A. Botrugno, Giovanni Tonon, Eduardo Sommella, Pietro Campiglia

**Affiliations:** 1 Department of Pharmacy, University of Salerno, Via Giovanni Paolo II, 84084 Fisciano, SA Italy; 2 PhD Program in Drug Discovery and Development, University of Salerno, Fisciano, SA Italy; 3 Department of Advanced Medical and Surgical Sciences, University of Campania “Luigi Vanvitelli”, 80138 Naples, Italy; 4 Functional Genomics of Cancer Unit, Division of Experimental Oncology, IRCCS San Raffaele Scientific Institute, Milan, Italy; 5 Vita-Salute San Raffaele University, Milan, Italy; 6 Center for Omics Sciences, IRCCS San Raffaele Scientific Institute, Milan, Italy

**Keywords:** Mass spectrometry, Metabolomics, Microbore, UHPLC, Untargeted

## Abstract

Untargeted metabolomics UHPLC-HRMS workflows typically employ narrowbore 2.1-mm inner diameter (i.d.) columns. However, the wide concentration range of the metabolome and the need to often analyze small sample amounts poses challenges to these approaches. Reducing the column diameter could be a potential solution. Herein, we evaluated the performance of a microbore 1.0-mm i.d. setup compared to the 2.1-mm i.d. benchmark for untargeted metabolomics. The 1.0-mm i.d. setup was implemented on a micro-UHPLC system, while the 2.1-mm i.d. on a standard UHPLC, both coupled to quadrupole-orbitrap HRMS. On polar standard metabolites, a sensitivity gain with an average 3.8-fold increase over the 2.1-mm i.d., along with lower LOD (LOD_avg_ 1.48 ng/mL vs. 6.18 ng/mL) and LOQ (LOQ_avg_ 4.94 ng/mL vs. 20.60 ng/mL), was observed. The microbore method detected and quantified all metabolites at LLOQ with respect to 2.1, also demonstrating good repeatability with lower CV% for retention times (0.29% vs. 0.63%) and peak areas (4.65% vs. 7.27%). The analysis of various samples, in both RP and HILIC modes, including different plasma volumes, dried blood spots (DBS), and colorectal cancer (CRC) patient-derived organoids (PDOs), in full scan-data dependent mode (FS-DDA) reported a significant increase in MS1 and MS2 features, as well as MS/MS spectral matches by 38.95%, 39.26%, and 18.23%, respectively. These findings demonstrate that 1.0-mm i.d. columns in UHPLC-HRMS could be a potential strategy to enhance coverage for low-amount samples while maintaining the same analytical throughput and robustness of 2.1-mm i.d. formats, with reduced solvent consumption.

**Supplementary Information:**

The online version contains supplementary material available at 10.1007/s00216-024-05588-z.

## Introduction

The growing interest in metabolomics has fueled the rise of metabolic phenotyping [1], which involves the comprehensive analysis of metabolites in biological fluids. The combination with other omics such as proteomics and genomics is driving the shift towards a patient phenotype-centric model, commonly referred to as personalized or precision medicine. Differently from proteomics and genomics, metabolomics analysis cannot be performed with a single analytical platform. This is related to the extreme chemical complexity and wide dynamic range of metabolites in biological fluids, cells, and tissues. Conventionally, nuclear magnetic resonance (NMR) spectroscopy; gas chromatography-mass spectrometry (GC‑MS); and liquid chromatography-mass spectrometry (LC‑MS) are employed, often in combination, to identify and quantify metabolites; each of these techniques has its own strength and weakness [2]. Nevertheless, LC–MS has become the technology of choice for metabolomics analysis, for its flexibility and sensitivity, and for the availability of multiple chromatography modes such as reversed phase (RP), hydrophilic interaction chromatography (HILIC), normal phase (NP), and supercritical fluid chromatography (SFC), which is extremely useful to handle the chemical and structural diversity of metabolite classes [3]. Additionally, the employment of columns packed with sub-2-μm particles in ultra-high-pressure conditions (UHPLC), combined with the accuracy, sensitivity, and fast acquisition times of novel mass analyzers, has certainly boosted the capabilities of LC–MS [4]. Still, one of the key challenges in metabolomics remains managing the vast dynamic range of the metabolome, which can vary significantly depending on the type and amount of sample, that in addition can differ in volume and availability. Conventionally, the preferred column diameter in LC–MS setups is narrowbore (2.1 mm) operated with analytical flow liquid chromatography (300–600 μL/min) [5]. A potential strategy to enhance the sensitivity of LC approaches involves reducing the internal diameter of the column. This reduction minimizes chromatographic dilution, thereby increasing sensitivity [6]. In the context of metabolomics, the consequent hyphenation with mass spectrometry could theoretically lead to the annotation and quantification of a higher number of metabolites. This aspect can be an important factor when restricted amounts of samples are available such as 3D cell models (e.g., spheroids and organoids, < 10E^4^ cells) and dried blood spots (5–10 µL), as well as in pharmaco-metabolomics and toxicological studies involving mice models, where the sampling process is crucial but can often cause pain and stress to the animals [7]. Additionally, reducing column diameter lowers solvent consumption and waste production, which is a key point for sustainability, particularly in large-scale studies. Nano-flow liquid chromatography-mass spectrometry (nLC-MS) is the cornerstone in proteomics; recently, the renaissance of microflow has gained popularity especially using 1.0-mm i.d. columns, resulting in very high throughput, robustness, and excellent identification rates [8, 9]. On the contrary, 1.0-mm i.d. columns have been scarcely used in metabolomics analysis, even if different authors have reported several benefits such as reduced sample requirements, lower solvent consumption, and sensitivity gain, since a concentration factor of 4.4 can be obtained with respect to standard 2.1-mm formats, if the same amount is injected on column [10, 11]. The limited use of 1.0-mm i.d. columns has been attributed to several factors: reduced loading capacity compared to 2.1-mm i.d. columns, lower packing efficiency, and the significant impact of extra-column band broadening in standard UHPLC systems. In this paper, we aimed to assess the performance of microbore columns in untargeted metabolomics and compared it with the 2.1-mm i.d. format. The objective of the work is to evaluate the use of 1.0-mm i.d. columns in UHPLC-HRMS-based metabolomics workflows across various sample types, including plasma, cells, organoids, and dried blood spots. A microflow and analytical flow system will be used, and both RP-UHPLC and HILIC conditions will be explored in conjunction with HRMS. The results will demonstrate the benefits of using 1.0-mm i.d. columns in untargeted analyses, offering a practical alternative to the standard 2.1-mm i.d. format.

## Materials and methods

### Chemicals

LC–MS-grade water (H_2_O), acetonitrile (ACN), methanol (MeOH), methyl tert-butyl ether (MTBE), LC–MS-grade additives formic acid (HCOOH), ammonium fluoride (NH_4_F), acetic acid (CH_3_COOH), and ammonium acetate (CH_3_COONH_4_) were purchased from VWR (Milan, Italy). Deuterium-labeled standards (L-Carnitine-d_9_, Taurine-d_9_, L-Tryptophan-d_5_, L-Lysine d_3_, L-Glutamate-d_5_, Taurohycholic Acid-d_4_, Butyric Acid-d_7_, Succinic Acid-d_4_) were purchased from Cayman Chemicals (Ann Arbor, MI, US) while authentic standards 2′-Deoxyadenosine-5′-monophosphate, 7-Methylguanine, Adenosine monophosphate (AMP), Alanine, Arginine, Asparagine, Asymmetric Dimethyl Arginine (ADMA), Bilirubin, Creatine, Glutamic acid, Glutamine, Glutathione RED, Histidine, Indolebutyric acid, Kynurenine, L-Alany-L-glutamine, L-Alanyl-L-phenylalanine, L-Carnitine, L-Carnosine, L-Citrulline, L-Homocitrulline, L-Pyroglutamic acid, Tryptophan, Lysine, Methionine, N^4^-Acetylcytidine, Nicotinamide Adenine Dinucleotide (NAD), Ornithine, Phenylalanine, Proline, Serine, Threonine, Thymine, Tyrosine, Uric acid, and Urocanic Acid were purchased from KEMA Science (Sorrento, Italy). Unless stated otherwise, other reagents were all purchased from Merck.

### Metabolome extraction from plasma, dried blood spots, and organoids

#### Plasma metabolome extraction

Two aliquots of human plasma of 2 and 20 µL (*n* = 3) from a pooled quality control sample made by using different aliquots of plasma belonging to healthy individuals were thawed on ice and extracted with 20 µL or 200 µL, respectively, of ice-cold MeOH/H_2_O 80:20 (v/v) containing a mixture of deuterated standards (Table S1). The samples were then vortexed for 12 min and incubated at − 20 °C for 30 min. They were then centrifuged at 19,275 rcf, for 10 min at 4 °C. Subsequently, the supernatants were collected and evaporated using a Speedvac (Savant, Thermo Scientific, Milan, Italy). The dried samples were dissolved in 50 µL of ACN/H_2_O 70:30 (v/v) and MeOH/H_2_O 10:90 (v/v), before HILIC or RP, respectively. The study protocol was approved by the local Ethics Committee (prot./SCCE no. 71262, May 2020). All methods and experimental procedures were performed under the Declaration of Helsinki.

#### Dried blood spot (DBS) metabolome extraction

Whole blood was collected from a healthy volunteer among the authors (*n* = 5 spots) (10.0 ± 0.5 µL) by using HemaXis DB10 (DBS Systems SA, Gland, Switzerland) following the manufacturer’s instructions, and then dried at room temperature overnight. Subsequently, DBS samples were added to 300 µL of ice-cold MeOH/H_2_O 80:20 (v/v) containing a mixture of deuterated standards. The samples were then vortexed for 12 min and incubated at − 20 °C for 30 min. They were then centrifuged at 19,275 rcf for 10 min at 4 °C. The supernatants were collected and evaporated using a Speedvac (Savant, Thermo Scientific, Milan, Italy). The dried samples were solubilized as reported previously.

#### Patients derived organoid (PDO) metabolome extraction

Non-treated (*n* = 3) and chemotherapy-treated (*n* = 3) PDO samples were provided by Functional Genomics of Cancer Unit of San Raffaele. Samples were extracted following an MTBE-based extraction. Samples were transferred from a 96-well plate to the tube and added to 225 µL of ice-cold MeOH containing a mix of deuterated standards. Subsequently, 750 µL of cold MTBE was transferred to the tube and the solution was continuously shaked in a thermomixer (Eppendorf, Milan, Italy) for 1 h at 4 °C. Subsequently, 188 µL of H_2_O was added and the samples were put on a vortex for 20 s and finally centrifuged at 19,275 rcf, for 10 min at 4 °C to induce phase separation. Finally, the lower phase containing polar metabolites was collected and evaporated using a Speedvac (Savant, Thermo Scientific, Milan, Italy). The dried samples were dissolved as reported for the previous matrices.

### Instrumentation and LC setup

Metabolome analyses were performed on a Thermo Vanquish Neo nano/micro UHPLC (1.0-mm i.d. setup) or a Vanquish Flex UHPLC (2.1-mm i.d. setup); each LC system was coupled online to two separate hybrid quadrupole Orbitrap Exploris 120 mass spectrometers (Thermo Fisher Scientific, Bremen, Germany) both equipped with a heated electrospray ionization probe (HESI II). For RP analyses, the separation was performed with an Acquity UPLC HSS T3™ (150 × 2.1 mm or 1.0 mm for the micro-setup; 1.8 µm, 100 Å) protected with a VanGuard HSS T3™ precolumn (5.0 × 2.1 mm; 1.8 µm, 100 Å) (Waters, Milford, MA, USA). For HILIC analyses, the separation was performed with an Acquity UPLC BEH Amide™ (100 × 2.1 mm or 1.0 mm for the micro-setup; 1.7 µm, 130 Å) protected with a VanGuard BEH Amide™ precolumn (5.0 × 2.1 mm; 1.7 µm, 130 Å) (Waters, Milford, MA, USA).

#### Microbore setup

For RP analyses, the column temperature was set at 55 °C, a flow rate of 100 µL/min was used, and mobile phases consisted of (A) H_2_O + 0.1% HCOOH and (B) ACN + 0.1% HCOOH were used for positive ionization, while 0.1% CH_3_COOH or 1 mM NH_4_F was used for negative ionization mode. The following gradient has been used: 0 min, 0% B; 6 min, 70% B; 8 min, 80% B; 9 min, 98% B, 10 min 98% B; 10.1 min, 0% B; and 3.9 min for column re-equilibration. For HILIC analyses, the column temperature was set at 55 °C, a flow rate of 90 µL/min was used, and mobile phases consisted of (A) H_2_O + 0.1% HCOOH or 95/5 H_2_O/ACN (v/v) + 10 mM CH_3_COONH_4_ and (B) ACN + 0.1% HCOOH or 95/5 ACN/H_2_O (v/v) + 10 mM CH_3_COONH_4_ were used for positive and negative ionization. The following gradient has been used: 0–0.1 min, 99% B; 7–7.7 min, 30% B; 7.8 min, 99% B; and 3.4 min for column re-equilibration. All the connections were nanoViper of 50-µm i.d. as standard Vanquish neo MicroLC configuration. An external oven was used and manually controlled (Phenomenex, Bologna, Italy).

#### Narrowbore setup

For RP mode, separation was carried out with a HSS T3 column (150 × 2.1 mm; 1.8 μm), protected with a Vanguard precolumn (5 × 2.1 mm; 1.7 μm) (Waters, Milan, Italy). The column temperature was set at 55 °C, and the flow rate was 500 µL/min. RP mobile phase was the same as reported for the microbore setup. The following gradient has been used: 0 min, 0% B; 1 min, 0% B; 1.5 min, 25% B; 4 min, 75% B; 6 min, 80% B; 6.1 min, 98% B; 7.1 min, 98%B; and 3.9 min for column re-equilibration. For HILIC mode, separation was carried out with a BEH Amide column (100 × 2.1 mm; 1.7 μm) protected with a Vanguard precolumn (5 × 2.1 mm; 1.7 μm) (Waters, Milan, Italy). The column temperature was set at 45 °C, and the flow rate was 0.400 mL/min. HILIC mobile phase was the same as reported for the microbore setup. The following gradient was employed: 0–0.1 min, 99% B; 0.1–8 min, 99–50% B; 8.0–8.5 min, 50–30% B; 8.5–9.5 min isocratic at 30% B; returning to 99% in 0.1 min, and then 4 min to recondition the column.

#### HRMS parameters

MS data acquisition for both setups was performed in full scan-data dependent acquisition (FS-DDA) in the m/z range 70–800. MS1 scan OT resolution, 60,000; AGC, auto; maximum injection time, 100 ms. S-Lens RF level, 70; ddMS2 OT resolution, 15,000; isolation window, 1.5 Da; dynamic exclusion, 10 s; AGC, auto; maximum injection time, 22 ms. TopN, 4; HCD fragmentation normalized collision energies (NCE): 20, 40, 60. The HESI source parameters for 1.0-mm i.d. setup were as follows: sheath gas, 20 a.u.; auxiliary gas, 7 a.u.; sweep gas, 0 a.u. Spray voltages were set to 3.3 kV and 3.0 kV for ESI (+) and ESI (−) respectively. Ion transfer tube and vaporizer temperatures were set to 280 °C and 150 °C respectively. The instrument was externally calibrated daily with FlexMix solution (ThermoFisher) while at the beginning of every LC run the internal calibrant was injected (IC run start mode). For 2.1-mm i.d. setup, source parameters were as follows: sheath gas pressure, 40 a.u. and 50 a.u. for positive and negative ionization modes, respectively; aux gas flow, 15 a.u.; sweep gas flow, 0 a.u. Spray voltages were set to 3.3 kV and 3.0 kV for ESI (+) and ESI (−). Ion transfer tube (ITT) and vaporizer temperatures were set to 300 °C and 320 °C. The same MS and MS/MS acquisition parameters and calibration were used as reported above.

### Data processing and statistical analysis

FreeStyle (Thermo Fisher Scientific) was used to visualize RAW data, which were then imported to Compound Discoverer v.3.3 (Thermo Fisher Scientific) to normalize, align, detect, and identify compounds. Features were extracted from 0–10 min and 0–11 min of the HILIC and RP chromatography runs, respectively, in the m/z = 70–800 mass range. Data were aligned according to an adaptive curve alignment model. Compounds were detected using the following parameters settings: mass tolerance was set to 5 ppm, while retention time tolerance was set to 0.2 min; minimum peak intensity was set to 100,000 AU and the signal to noise threshold for compound detection was set to 5. The peak rating filter was set to 3. To perform blank subtraction, we maintained max sample/max blank ratio > 5. For statistical comparison between the two setups, signal intensity was normalized by using the algorithm “Constant Sum.” To predict elemental compositions of the compounds, the relative intensity tolerance was set to 30% for isotope pattern matching. For the mzCloud database search, both the precursor and fragment mass tolerance were set to 5 ppm. The databases used for matching compounds in ChemSpider for structural search were BioCyc, the Human Metabolome Database, and KEGG, and the mass tolerance in ChemSpider Search was set to 5 ppm. The mass tolerance for matching compounds in Metabolika pathways was set to 5 ppm. Compounds were assigned by comparing annotations using the following nodes in order of priority: (1) mzCloud; (2) Predicted Compositions; (3) MassList search; (4) ChemSpider Search; (5) Metabolika search. Principal component analysis was performed by MetaboAnalyst 6.0 (https://www.metaboanalyst.ca/); samples were log transformed and autoscaled prior to statistical analysis, all other graphs were built using GraphPad Prism 8.0 (GraphPad Software, Boston, MA, USA, www.graphpad.com).

## Results and discussion

### Optimization of 1.0-mm i.d.-based approach

**Fig. 1:**
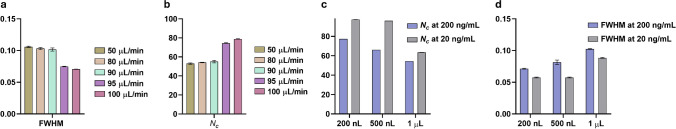
**a**–**d** Effect of flow rate and injection volume on FWHM and peak capacity in RP mode

To compare the performance of microbore and narrowbore setups, we used columns of the same length, stationary phase chemistry, and particle size using a nano/micro-LC system and an equivalent analytical flow UHPLC system, respectively. No hardware modifications were made to the microbore system, which already had 50-µm i.d. fluidics, including inlet and outlet column tubing. In the standard flow UHPLC system, all fluidic connection tubing remained at 0.1-mm i.d.. To benchmark the performance of 1.0-mm column against the 2.1-mm i.d., a mixture of endogenous metabolite standards was used (Table S1); LC method for 2.1-mm column was based on in-house previously optimized method [12] with slight modifications. The effects of flow rate, injection volume, concentration, and gradient length were assessed with endogenous standards injected at two concentration levels on the 1.0-mm i.d. column, evaluating peak width at half maximum (FWHM) and subsequently peak capacity. Initially, we investigated various flow rates (50, 80, 90, 95, and 100 μL/min). As expected, and as illustrated in Fig. 1a–d, peak width decreased with increasing flow rate, reaching the lowest value of 0.14 min at 100 μL/min in RP mode.

Conversely, for the investigated metabolites, higher values were observed in HILIC mode (0.19 min, Fig. S1a–d). Although HILIC is orthogonal to RP and capable of higher retention of the polar metabolome, it typically yields larger peak widths [13]; additionally, it must be noted that the employed RP column was longer than the HILIC (150 vs. 100 mm).

Peak capacity values were calculated as follows [14]:

$${n}_{c}=1+\frac{Tg}{w}$$

In agreement with the overall reduction of peak width, the highest *n*_c_ values for the 1.0-mm i.d. were obtained at 100 μL/min in RP mode, *n*_c_ = 78 (Fig. 1b). The method transfer from the 2.1-mm i.d. column would be translated in a flow rate (F1 to F2) of 113 μL/min, calculated with the gradient transfer method calculator [15] (https://farma-unites.unige.ch/en/rudaz-lab/tools/hplc-calculator). Nevertheless, we were constrained by the upper flow rate limit of the Vanquish Neo system (100 μL/min), which was further reduced at 90 μL/min in HILIC mode since the pressure limit of the system was reached, resulting in the lower value of *n*_c_ = 47 (Fig. S1b). In this regard, we tried to higher flow rates (up to 120 μL/min) by installing the 1.0-mm i.d. column on the analytical UHPLC system, but the obtained results were considerably lower (− 48.38%) than the values obtained on the microflow system (Table S2), thus enforcing the importance of extra-column band broadening in using 1.0-mm i.d. columns on standard UHPLC systems. Next, we investigated the impact of injection volume and sample concentration. Injection volume and resulting column overload are significant challenges in microbore columns. Consequently, 0.2, 0.5, and 1 μL injections were tested at two concentration levels, 200 and 20 ng/mL, respectively. The best results were obtained with an injection volume of 200 nL (Fig. 1c) using a sample concentration of 20 ng/mL (*n*_c_ = 97) (Fig. 1d, Fig. S1d). Clearly, these results were obtained on standard compounds, while decreasing the injection volume improves chromatographic efficiency, it’s imperative to find a balance with sensitivity, as the identification of low-abundance metabolites is essential for untargeted approaches [16]. Lastly, a comparison of different gradient lengths was performed; Fig. S2 highlights that the 30-min gradient condition nearly doubles the peak capacity values (*n*_c_ = 141 for RP, *n*_*c*_ = 90 for HILIC) as expected for peak width compression, in agreement with the evidence that increasing the gradient times generally results in higher peak capacity, even if for small molecule compounds with longer gradient times, the peak capacity tends to a limiting value [17]. Moreover, it should be noted that analysis time is a critical aspect in untargeted metabolomics, especially when dealing with a high number of samples. Considering these factors and aiming to maintain equivalent throughput between the 2.1-mm and 1.0-mm i.d. setups, we opted for the shorter gradient condition for direct comparison with the 2.1-mm i.d. method, and equal injection volume and concentration. Overall, we compared the chromatographic performance to the 2.1-mm i.d.; in this regard and as expected, the results indicated that the performance of the 1.0-mm i.d. methods was still lower than that of 2.1 mm (FWHM 0.1 min vs. 0.14 min, *n*_c_ 103 vs. 78, Fig. S3). This reduction, as previously reported, can be attributed to operating the 1.0-mm i.d. columns slightly below the recommended flow rate due to the upper flow constraints of the micro-LC system employed, along with potential additional band broadening at the MS source. In this context, it must be noted that we opted to utilize the high-flow HESI capillary for enhanced robustness and reduced risk of clogging when analyzing real samples.

### Sensitivity comparison between 1.0-mm and 2.1-mm i.d. setups on polar standard metabolites

**Fig. 2:**
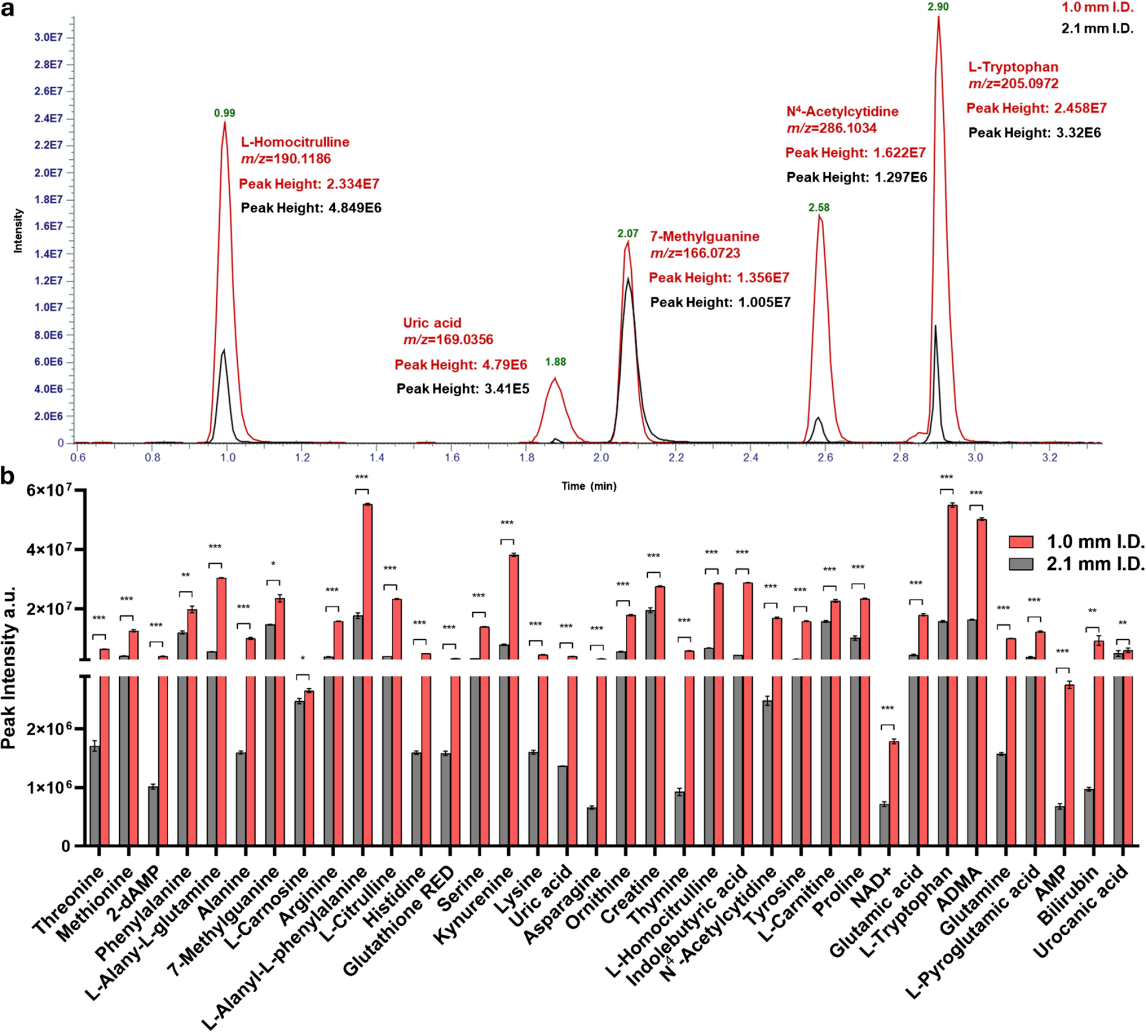
**a** EICs of metabolite standards, L-Homocitrulline, Uric acid, 7-Methylguanine, N^4^-acetylcitidine, and L-Tryptophan between 1.0-mm (red) and 2.1-mm i.d. (black). **b** Comparison of peak intensity between 1.0- and 2.1-mm i.d. methods for polar standard metabolites

Peak intensities for the investigated standard metabolites were then compared between the 2.1-mm i.d. setup and the 1.0-mm i.d. with the conditions reported previously. By comparison of metabolite peak intensities, as can be observed from Fig. 2a, an average fold change (FC_avg_) intensity gain of 3.79 was obtained. The highest value was observed for N4-Acetylcytidine (FC, 6.86) while the lowest for L-Carnosine (FC, 1.07) in RP(^+ / −^) mode. HILIC-ESI(^+ / −^) showed generally lower FC values (FC_avg_, 1.48), with homocitrulline showing the highest value (FC, 3.04) and bilirubin showing the lowest (FC, 0.22).

**Fig. 3:**
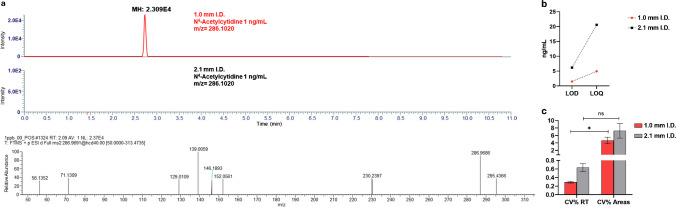
**a** Extracted ion chromatogram of N.^4^-acetylcitidine at 1 ng/mL and MS/MS spectrum showing no precursor detected in the 2.1-mm I.D. method. **b** LOD and LOQ comparison between 1.0 (red) and 2.1 (black). **c** Repeatability comparison between 1.0- (red) and 2.1-mm i.d. (black)

The differences in response were compound dependent. However, as illustrated in the extracted ion chromatograms (EICs) in Fig. 2b, they were also correlated with metabolite retention in both modalities. In fact, metabolites with longer retention times demonstrated a more substantial increase in peak intensity compared to early-eluting metabolites. Only slight differences in retention times across the two LC systems are observed (± 16%), which denotes no significant additional delay volume of the micro-LC system. Since untargeted metabolomics often faces profound differences of analyte concentrations in biospecimens, we then moved to compare the performance of the 1.0-mm i.d. method in terms of dynamic range, by preparing calibration curves of standard metabolites spanning three orders of magnitude (Fig. S4). While linearity was essentially similar and satisfying in both methods, on average, the 1.0-mm i.d. method was able to detect and quantify all the investigated analytes even at the lowest concentration, on the contrary, the 2.1-mm i.d. method was unable to detect 10/15 of the investigated metabolites in the lower concentration range (1–5 ng/mL). This can be immediately appreciated in Fig. 3a, showing the EICs of metabolite N^4^-Acetylcytidine at the LLOQ. As can be observed, the metabolite is still detected and quantified in the 1.0-mm i.d. method, resulting in the selection and isolation of its precursor in DDA and acquisition of the corresponding MS/MS spectrum. Contrariwise, the 2.1 mm i.d. method was unable to detect the metabolite at this concentration. In this regard, we moved to compare the limit of detection (LOD) of the two approaches as follows:

$$\text{LOD}= 3 \times \frac{Sd}{b}$$

where *s* is the residual standard deviation of the calibration line in the LOD region and *b* is calibration graph slope. The results showed that the 1.0-mm i.d. on average possesses twofold lower limit of detection (LOD_avg_ 1.48 ng/mL vs. 6.18 ng/mL). Similar results were obtained for the limit of quantification, which was calculated as follows:

$$\text{LOD}= 10 \times \frac{Sd}{b}$$

In this regard, almost threefold lower values (LOQ_avg_ 4.94 ng/mL vs. 20.60 ng/mL) can be appreciated from Fig. 3b. These data are compound dependent and can be influenced by the reproducibility of peak area, but it is evident how the microbore setup possesses lower values. Complete data for the remaining metabolite standards employed in the optimization phase are reported in Table 1.

**Table 1:** Complete data for the remaining metabolite standards employed in the optimization phase

Endogenous standard	Linear range ng/mL	Coefficient of correlation (*R*^2^)	Limit of detection (LOD) ng/mL	Limit of quantification (LOQ) ng/mL	Repeatability		Accuracy
					CV% RT	CV% areas	
Microbore setup							
2′-Deoxyadenosine-5′-monophosphate	5–1000 ng/mL	*R*^2^ = 0.996	1.742	5.805	0.46%	5.23%	**96.66%**
2′-Deoxyuridine	1–1000 ng/mL	*R*^2^ = 0.999	3.025	10.085	0.00%	0.71%	**95.88%**
2-Oxoadipic acid	10–1000 ng/mL	*R*^2^ = 0.999	4.219	14.064	0.01%	0.01%	**88.96%**
7-Methylguanine	1–1000 ng/mL	*R*^2^ = 0.999	0.213	0.710	0.00%	6.73%	**90.27%**
Creatine	1–1000 ng/mL	*R*^2^ = 0.999	0.256	0.854	0.00%	5.90%	**87.13%**
Guanine	25–1000 ng/mL	*R*^2^ = 0.999	1.027	3.424	0.00%	0.06%	**91.68%**
Kynurenine	5–1000 ng/mL	*R*^2^ = 0.999	0.875	2.918	0.23%	9.80%	**97.45%**
L-Carnitine	5–1000 ng/mL	*R*^2^ = 0.999	0.452	1.506	0.71%	7.90%	**88.68%**
L-Carnosine	5–1000 ng/mL	*R*^2^ = 0.998	0.282	0.941	0.82%	9.26%	**84.09%**
L-Homocitrulline	5–1000 ng/mL	*R*^2^ = 0.997	0.891	2.970	0.00%	9.42%	**88.18%**
L-Tryptophan	1–1000 ng/mL	*R*^2^ = 0.999	1.989	6.631	0.20%	5.77%	**99.18%**
N^4^-Acetylcytidine	1–1000 ng/mL	*R*^2^ = 0.999	0.703	2.344	0.23%	8.24%	**88.18%**
Thymine	5–1000 ng/mL	*R*^2^ = 0.999	2.005	6.682	0.00%	6.44%	**98.07%**
Urocanic acid	1–1000 ng/mL	*R*^2^ = 0.999	3.083	10.277	0.00%	0.01%	**93.19%**
Narrowbore setup							
2′-Deoxyadenosine-5′-monophosphate	10–1000 ng/mL	*R*^2^ = 0.999	2.905	9.683	0.31%	3.79%	**91.73%**
2′-Deoxyuridine	25–1000 ng/mL	*R*^2^ = 0.999	4.240	14.140	1.02%	2.05%	**91.75%**
2-Oxoadipic acid	10–1000 ng/mL	*R*^2^ = 0.999	4.760	15.860	0.82%	1.62%	**98.77%**
7-Methylguanine	1–1000 ng/mL	*R*^2^ = 0.999	0.265	0.884	0.34%	2.04%	**94.72%**
Creatine	5–1000 ng/mL	*R*^2^ = 0.999	0.250	0.835	0.72%	2.71%	**94.97%**
Guanine	25–1000 ng/mL	*R*^2^ = 0.999	7.315	24.384	0.37%	5.17%	**95.78%**
Kynurenine	10–1000 ng/mL	*R*^2^ = 0.999	7.679	25.595	0.00%	1.80%	**89.80%**
L-Carnitine	10–1000 ng/mL	*R*^2^ = 0.999	1.432	4.773	0.94%	9.19%	**86.60%**
L-Carnosine	10–1000 ng/mL	*R*^2^ = 0.997	0.800	2.666	0.94%	9.19%	**75.56%**
L-Homocitrulline	5–1000 ng/mL	*R*^2^ = 0.999	0.160	0.520	0.66%	5.87%	**89.97%**
L-Tryptophan	1–1000 ng/mL	*R*^2^ = 0.998	3.270	10.901	0.22%	2.43%	**90.01%**
N^4^-Acetylcytidine	50–1000 ng/mL	*R*^2^ = 0.989	8.265	27.550	1.02%	5.13%	**87.59%**
Thymine	50–1000 ng/mL	*R*^2^ = 0.997	44.455	148.184	0.42%	16.50%	**96.09%**
Urocanic acid	5–1000 ng/mL	*R*^2^ = 0.999	0.739	2.465	0.57%	1.62%	**96.41%**

Clearly, signal to noise ratio (*S*/*N*) values are improved on the 1.0-mm i.d. setup; in this regard, the lower flow rate used for the 1.0-mm i.d. column increases desolvation and ionization efficiency in ESI [18]. Complete data are reported in supplementary Table S3. Additionally, assessment of probe position as well as vaporizer temperature was also investigated to identify the most suitable conditions. The H-ESI source can be moved in different positions: *X* (side to side), *Y* (front to back), and *Z* (vertical). The different working positions in these three directions determine the proximity of the H-ESI probe to the ITT. We evaluated the effect of the *Z* positions by keeping the *X* direction at the center position and *Y* at 1. Our results revealed that a small increase in peak height can be obtained by moving the H-ESI source at low (L) *Z* position. Furthermore, we explored the potential impact of vaporization temperature on ionization by testing different vaporizer temperatures: 0 °C, 100 °C, 150 °C, and 200 °C. Our findings suggest that working at 150 °C vaporizer temperature may offer more favorable results; hence, the higher intensity was obtained with the probe position “L” and vaporizer temperature of 150 C° (Fig. S5).

### Robustness and repeatability of the 1.0-mm i.d. setup

In untargeted metabolomics studies, the repeatability of retention time and peak areas are critical aspects, especially during pre-processing steps such as peak alignment. In this regard, 1.0 mm i.d. showed lower intraday CV% values for both retention time and peak area when compared to 2.1-mm i.d. setup (Fig. 3c) (CV RT 0.29% vs. 0.63%, CV areas 4.65% vs. 7.27%), with similar values obtained for intraday (data not shown). Additionally, by using real samples, after 250 injections over 48 h of consecutive analyses, the system backpressure showed no signs of alteration as can be seen from the backpressure traces during the entire gradient, reported in Fig. S6. Complete data are reported in Table 1. Notably, the solvent consumption in the microbore setup over 250 runs was just 300 mL with respect to 1.5 L in the narrowbore setup.

### Metabolome coverage and comparison with 2.1-mm i.d. setup over different samples

**Fig. 4:**
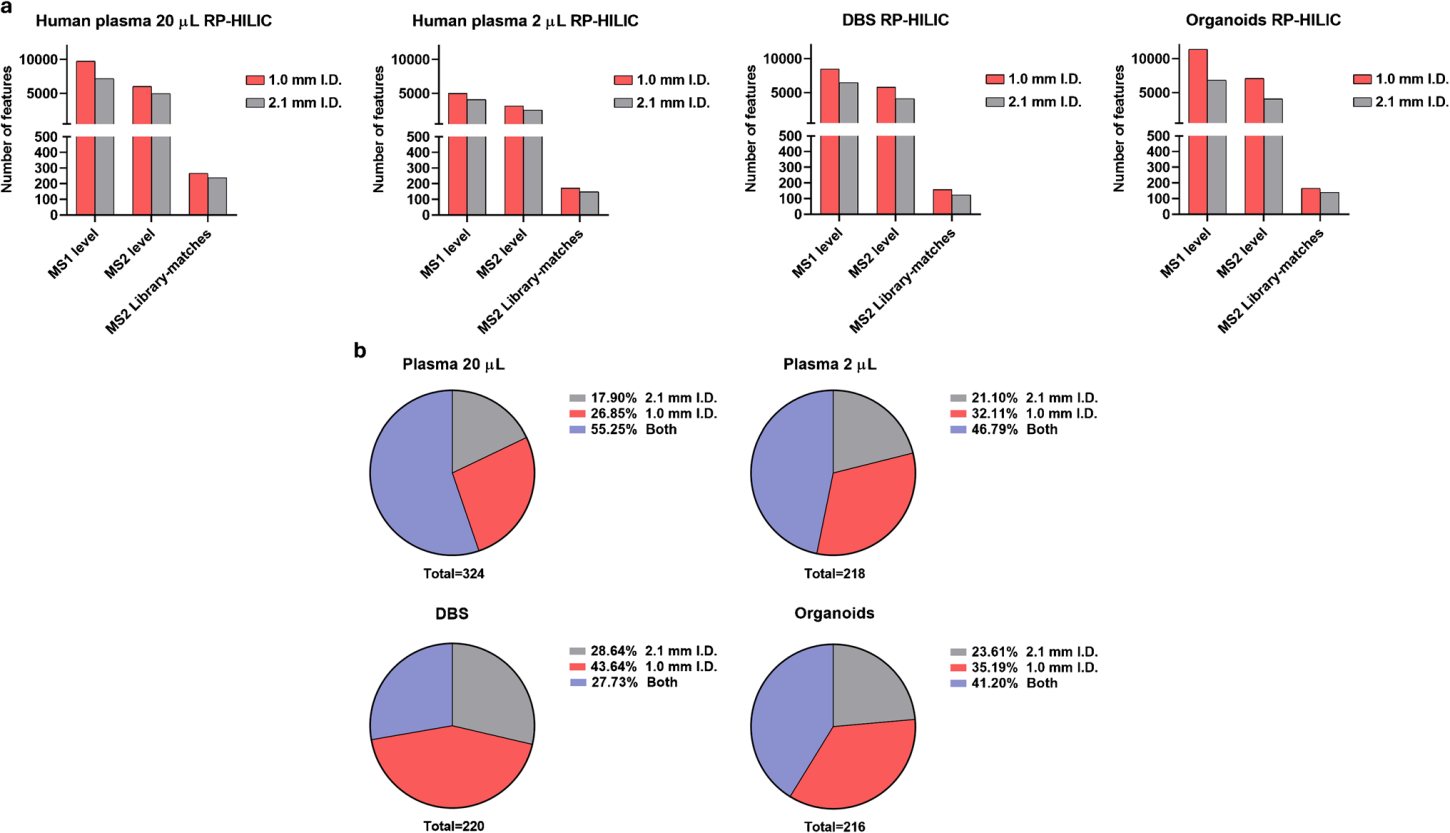
**a** Comparison of MS1, MS/MS, and MS/MS spectral library matches’ features between 1.0-mm (red) and 2.1-mm i.d. (gray) setups. **b** Annotation overlap between the two approaches

**Fig. 5:**
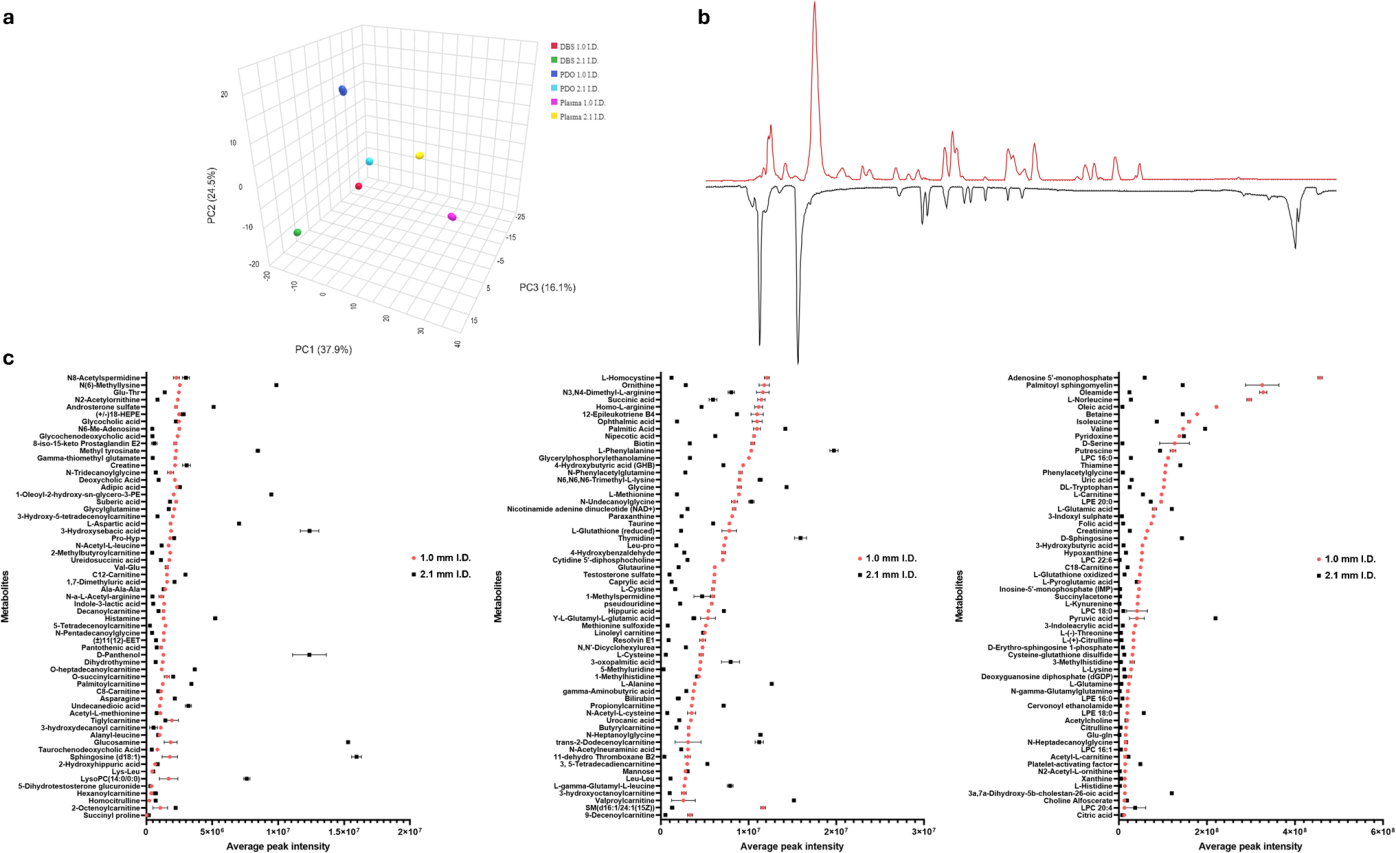
**a** Principal component analysis 3D score plot showing the distinct clustering of the four analyzed matrices between 1.0- and 2.1-mm i.d. approaches. **b** Base peak chromatograms of a PDO analyzed on the 1.0- (red) and 2.1-mm i.d. (black) setup. **c** Comparison of peak intensity for the shared annotations in the four investigated matrices between 1.0- and 2.1-mm i.d. methods on real samples

To further investigate the potential increase in metabolome coverage, we analyzed various samples: human plasma at two different volumes, dried blood spots, and patient-derived colorectal cancer organoids. Metabolites were annotated using MS/MS spectral libraries mzCloud and mzVault (MSI level 2), and when available, through direct comparison with authentic standards (MSI level 1) [19]. Each sample was analyzed using a combination of reversed-phase (RP) and hydrophilic interaction liquid chromatography (HILIC) methods, as their complementary use significantly increases metabolome coverage [20]. In this regard, we also tested different mobile phase additives, resulting in the best combination of formic acid and ammonium fluoride in RP for ESI ( +) and ( −) respectively, while ammonium acetate was selected in HILIC for both polarities (data not shown). Subsequently, we merged RP and HILIC annotations, at each MS level and polarity, removing duplicates and reporting only the adduct with the highest intensity, to express the effective gain of features in the 1.0-mm i.d. setup. Figure 4a reports the comparison in terms of percentage increase of MS1 features, MS2 features, and library-matching MS/MS spectra for each investigated matrix. As can be observed, the 1.0-mm i.d. was able to annotate on average more MS1 features (MS1_avg_ =  + 38.95%) and thus trigger more MS/MS events (MS/MS_avg_ =  + 39.26%); this can be visualized from the dot-plot maps in Fig. S7 showing the highest number of red dots corresponding to the precursor selected for HCD. Owing to the MS/MS library matches and standard comparison, clearly, the difference in the number of features is less than MS1 and MS/MS (MS/MS_Lib.match_avg_ =  + 18.23%). While lower than values observed for MS1 and MS2, this increase can be highly useful to detect low abundant features that can be crucial to identify modulated metabolites in key molecular pathways. Additionally, as recently underlined [21], the discrepancy between the unannotated and annotated MS/MS spectra, relies on the influence of the in-source fragmentation that occurs before the MS/MS event, thus resulting in many unidentified metabolites. As shown in Fig. 4b, on average for the four investigated matrices, over 34% of the metabolites were annotated only with the 1.0-mm i.d. method, thus enforcing its potential for untargeted approaches. Principal component analysis (PCA) score plot in Fig. 5a reports the grouping of the different biological matrices obtained by using the 1.0- and the 2.1-mm i.d.-based approaches. It is evident how the same sample differently clusters; this is clearly related to the distinct metabolic coverage provided by the two approaches, which underlines the capacity of the 1.0-mm i.d. to capture more metabolic features than narrowbore approach, which is immediately appreciable from the higher number of peaks that can be observed from the base peak chromatograms depicted in Fig. 5b, which reports the comparison of the same organoid extract analyzed on the two different setups by RP-ESI(-), resulting in an increase in the biological information that can be accessed. Overall, by merging the results for the four investigated matrices and considering multiple endogenous metabolites classes present, such as nucleotides, amino acids and derivatives, carnitines, and organic acids (Fig. 5c), characterized by both RP and HILIC, the average fold increase was 1.98. These results are in line with previous observations that compared 1.0- and 2.1-mm i.d. setups, but in that case, the authors only considered RP mode [22]. The merged metabolite annotations resulted in a global coverage of 507 annotated metabolites. In this regard, the 1.0-mm i.d. method showed higher intensity for 130/184 shared annotations, which are mainly represented by amino acids and derivatives, dipeptides, short-chain acylcarnitines, and fatty acid conjugates (Fig. S8a). On the contrary, 219 annotations were detected only with the 1.0-mm i.d. method, and the difference in coverage can be better appreciated by Fig. S8b, which clearly reports more metabolite subclasses. Concerning the 104 annotations that were detected only with the 2.1-mm i.d. method (Fig. S8c), these were mainly represented by glycerophospholipids, fatty acids, and long-chain acylcarnitines. A potential explanation is that since these compounds are at the interface between semi-polar and non-polar metabolites (lipids) and the employed H-ESI conditions in the 1.0-mm i.d. setup (such as ion spray voltage, gas temperatures, and pressure) could be not fully optimized and impact their ionization. This aspect is mainly related to the fact that in our approach H-ESI parameters were tuned by using only polar metabolites, which clearly possess different behaviors in terms of ionization. On the contrary, the H-ESI conditions for 2.1-mm i.d. were derived from well-established parameters at analytical flow rate on Orbitrap mass analyzers. Additionally, these compounds are usually highly retained in RP and are characterized by very narrow peak widths also delivered by the slightly higher peak capacity of the narrowbore approach, which in turn boost their response, also for low abundant compounds. Clearly, this represents a limitation of the 1.0-mm i.d. setup, whose LC–MS conditions have been optimized mainly using polar metabolites; this aspect underscores that additional work should be performed to obtain more coverage for additional compound classes, such as semi-polar and non-polar metabolites, that usually require different conditions of mobile and stationary phases. In fact, recent application showed the potential of microbore approaches for targeted lipidomics [23], by using typical LC conditions that are suited for lipids and non-polar metabolites.

## Conclusion

In this work, we have explored and demonstrated the utility of 1.0-mm i.d. microbore column-based separation for untargeted metabolomics. Higher signal intensity, with lower values of LOD and LOQ, is obtained, resulting in appreciable gain in the overall coverage for polar metabolome with respect to 2.1-mm i.d. setup, together with better repeatability and robustness in the analysis of real samples. The developed method shows an average 1.3-fold increase in response compared to conventional narrowbore setup for several biospecimens, while maintaining similar throughput of the 2.1-mm i.d. approach. The method is able to have more metabolome information across different matrices, from conventional to low amount. Finally, and noteworthy, a drastic reduction of solvent consumption is obtained. These results underline the potential employment of 1.0-mm i.d. microbore columns in untargeted metabolomics as a valuable alternative to narrowbore-based separations to achieve higher metabolome coverage and same analytical throughput. Further extension to non-polar metabolites and lipids could expand the utilization and coverage of 1.0-mm i.d. setup. Lastly, the drastic reduction of solvent consumption makes this approach environmentally friendly, especially when dealing with the screening of large cohorts of sample.

## Supplementary Information

Below is the link to the electronic supplementary material.

Supplementary file1 (DOCX 1878 KB)

Supplementary file2 (XLSX 28 KB)
