# Supplementary material for: Leveraging the potential of 1.0-mm i.d. columns in UHPLC-HRMS-based untargeted metabolomics
Source: Anal Bioanal Chem. 2024 Oct 24;417(13):2837–47. doi: 10.1007/s00216-024-05588-z (PMC12053354; doi:10.1007/s00216-024-05588-z)
Supplement: Supplementary file 1 — Supplementary file1 (DOCX 1878 KB) [file 216_2024_5588_MOESM1_ESM.docx]

**Leveraging the potential of 1.0 mm I.D. columns in UHPLC-HRMS based untargeted metabolomics**

Danila La Gioia^1,2*^, Emanuela Salviati^1*^, Manuela Giovanna Basilicata^3^, Claudia Felici^4^, Oronza A Botrugno^4,5^, Giovanni Tonon^4,5,6^, Eduardo Sommella^1^, Pietro Campiglia^1#^

^1^Department of Pharmacy, University of Salerno, Via Giovanni Paolo II, 13284084, Fisciano, SA, Italy.

^2^PhD Program in Drug Discovery and Development, University of Salerno, Fisciano, SA, Italy.

^3^Department of Advanced Medical and Surgical Sciences, University of Campania “Luigi Vanvitelli” , Naples, 80138, Italy

^4^Functional Genomics of Cancer Unit, Division of Experimental oncology, IRCCS San Raffaele Scientific Institute, Milan, Italy

^5^Vita-Salute San Raffaele University, Milan, Italy.

^6^Center for Omics Sciences, IRCCS San Raffaele Scientific Institute, Milan, Italy.

*These authors equally contribute as first author

^#^Corresponding author

| **Deuterated standards** | **Molecular formula** |
| --- | --- |
| Butyric Acid-d7 | C_4_HD_7_O_2_ |
| L-Carnitine-d9 | C_7_H_7_D_9_NO_3_.Cl |
| L-Glutamic Acid-d5 | C_5_H_4_D_5_NO_4_ |
| L-Lysine-d3 | C_6_H_11_D_3_N_2_O_2_.HCl |
| L-Tryptophan-d5 | C_11_H_7_D_5_N_2_O_2_ |
| Succinic Acid-d4 | C_4_H_2_D_4_O_4_ |
| Taurine-d4 | C_2_H_3_D_4_NO_3_S |
| Taurohycholic Acid-d4 | C_26_H_40_D_4_NO_7_S.Na |
| **Endogenous standards** | **Molecular formula** |
| 2‘-Deoxyadenosine-5’-monophosphate | C_10_H_14_N_5_O_6_P |
| 7-Methylguanine | C_6_H_7_N_5_O |
| Adenosine monophosphate (AMP) | C_10_H_14_N_5_O_7_P |
| Alanine | C_3_H_7_NO_2_ |
| Arginine | C_6_H_14_N_4_O_2_ |
| Asparagine | C_4_H_8_N_2_O_3_ |
| Asymmetric Dimethyl Arginine (ADMA) | C_8_H_18_N_4_O_2_ |
| Bilirubin | C_33_H_36_N_4_O_6_ |
| Creatine | C_4_H_9_N_3_O_2_ |
| Glutamic acid | C_5_H_9_NO_4_ |
| Glutamine | C_5_H_10_N_2_O_3_ |
| Glutathione RED | C_10_H_17_N_3_O_6_S |
| Histidine | C_6_H_9_N_3_O_2_ |
| Indolebutyric acid | C_12_H_13_NO_2_ |
| Kynurenine | C_10_H_12_N_2_O_3_ |
| L-Alany-L-glutamine | C_8_H_15_N_3_O_4_ |
| L-Alanyl-L-phenylalanine | C_12_H_16_N_2_O_3_ |
| L-Carnitine | C_7_H_15_NO_3_ |
| L-Carnosine | C_9_H_14_N_4_O_3_ |
| L-Citrulline | C_6_H_13_N_3_O_3_ |
| L-Homocitrulline | C_7_H_15_N_3_O_3_ |
| L-Pyroglutamic acid | C_5_H_7_NO_3_ |
| L-Tryptophan | C_11_H_12_N_2_O_2_ |
| Lysine | C_6_H_14_N_2_O_2_ |
| Methionine | C_5_H_11_NO_2_S |
| N^4^-Acetylcytidine | C_11_H_15_N_3_O_6_ |
| Nicotinamide Adenine Dinucleotide (NAD) | C_21_H_27_N_7_O_14_P_2_ |
| Ornithine | C_5_H_13_N_2_O_2_.Cl |
| Phenylalanine | C_9_H_11_NO_2_ |
| Proline | C_5_H_9_NO_2_ |
| Serine | C_3_H_7_NO_3_ |
| Threonine | C_4_H_9_NO_3_ |
| Thymine | C_5_H_6_N_2_O_2_ |
| Tyrosine | C_9_H_11_NO_3_ |
| Uric acid | C_5_H_4_N_4_O_3_ |
| Urocanic Acid | C_6_H_6_N_2_O_2_ |

**Table S1.** Deuterated internal standards and endogenous external standards

| **90 µL/min** | | | |
| --- | --- | --- | --- |
| 1.0 mm i.d. setup on microUHPLC | | 1.0 mm i.d. setup on analytical UHPLC | |
| FWHM | Peak Capacity | FWHM | Peak Capacity |
| 0.105 | 54.425 | 0.143 | 39.658 |
| **100 µL/min** | | | |
| 0.07 | 78.800 | 0.136 | 40.672 |

**Table S2.** FWHM and peak capacity values obtained by installing the 1.0 mm i.d. column on the analytical UHPLC system.


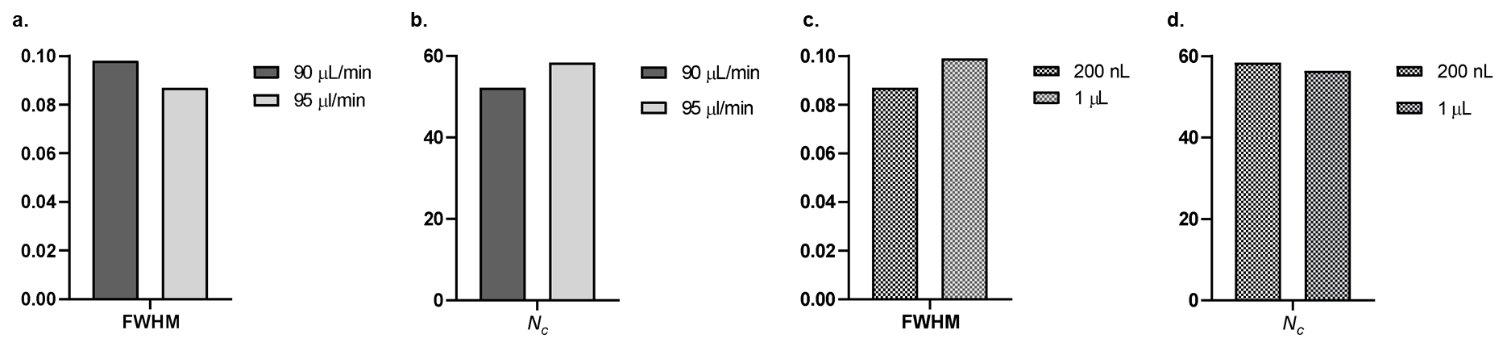


**Figure S1a-d:** Effect of flow rate and injection volume on FWHM and peak capacity in HILIC mode


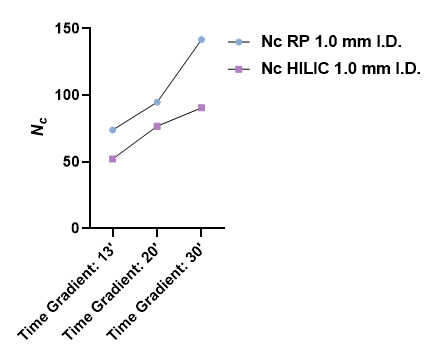


**Figure S2:** Effect of gradient length on peak capacity on both RP and HILIC


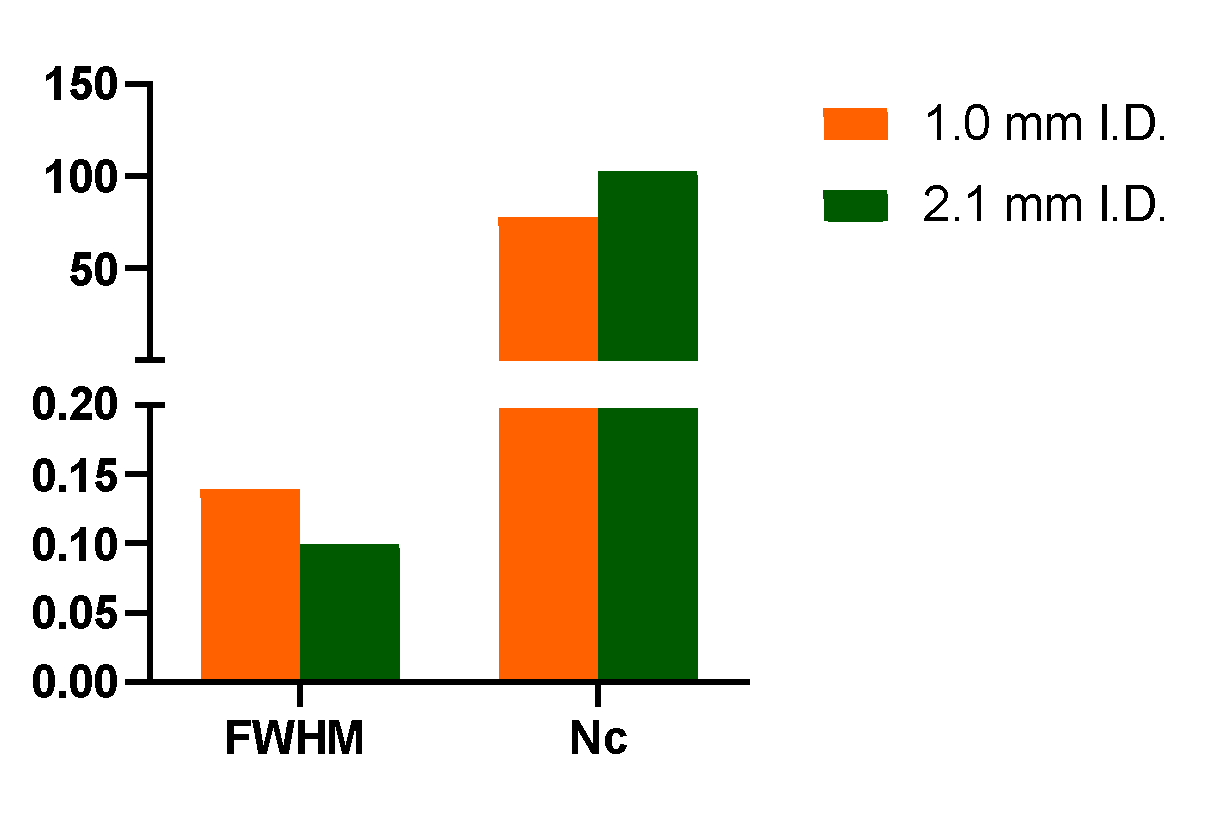


**Figure S3:** Final comparison of FWHM and peak capacity between 1.0 and 2.1 mm I.D setups


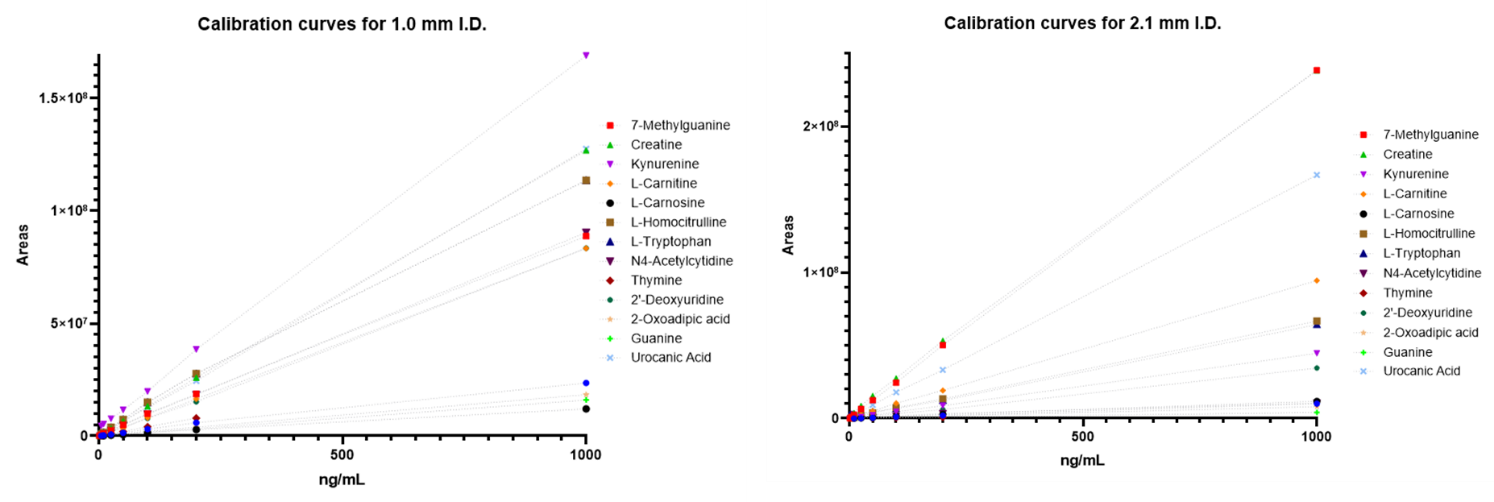


**Figure S4:** Calibration curves for 1.0 and 2.1 mm ID setup

**
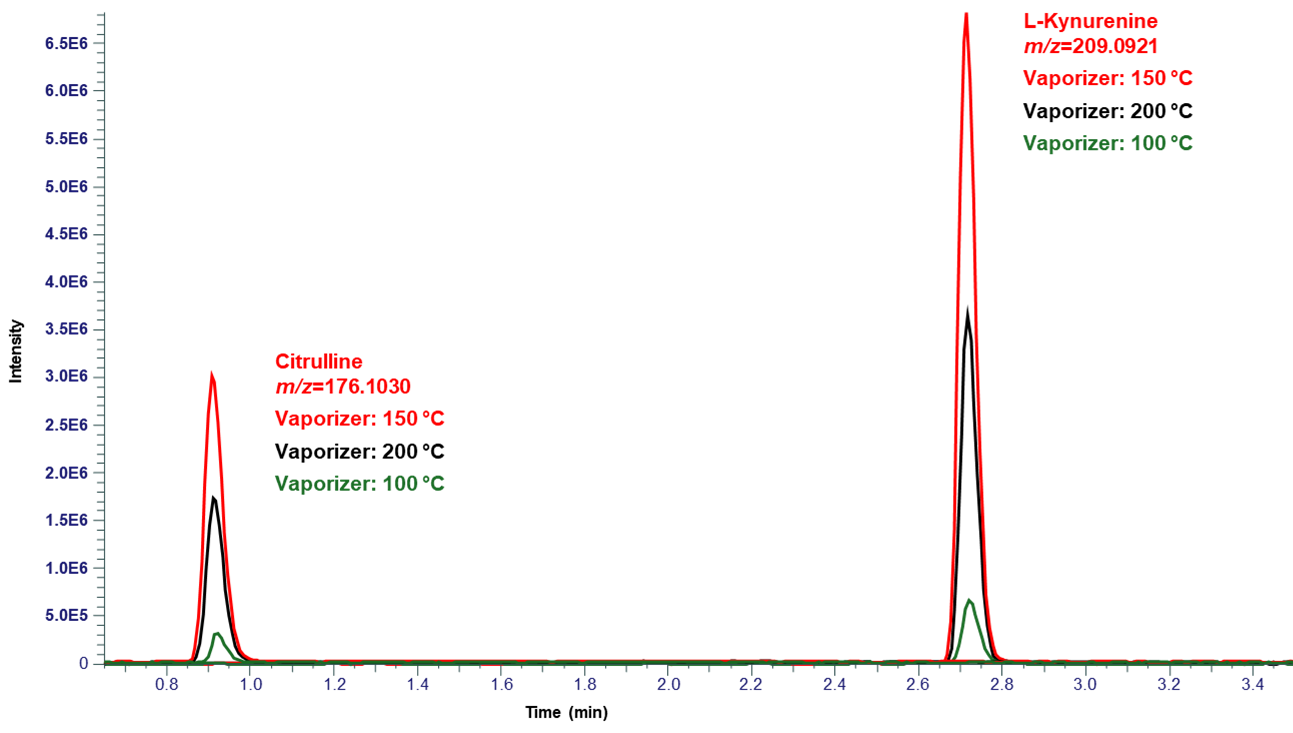
**

**Figure S5:** EICs of kynurenine *m/z*=209.0921 and citrulline *m/z=*176.1030 in positive ionization mode employing vaporizer temperature at 100 °C in green, 150 °C in red and 200 °C in black.


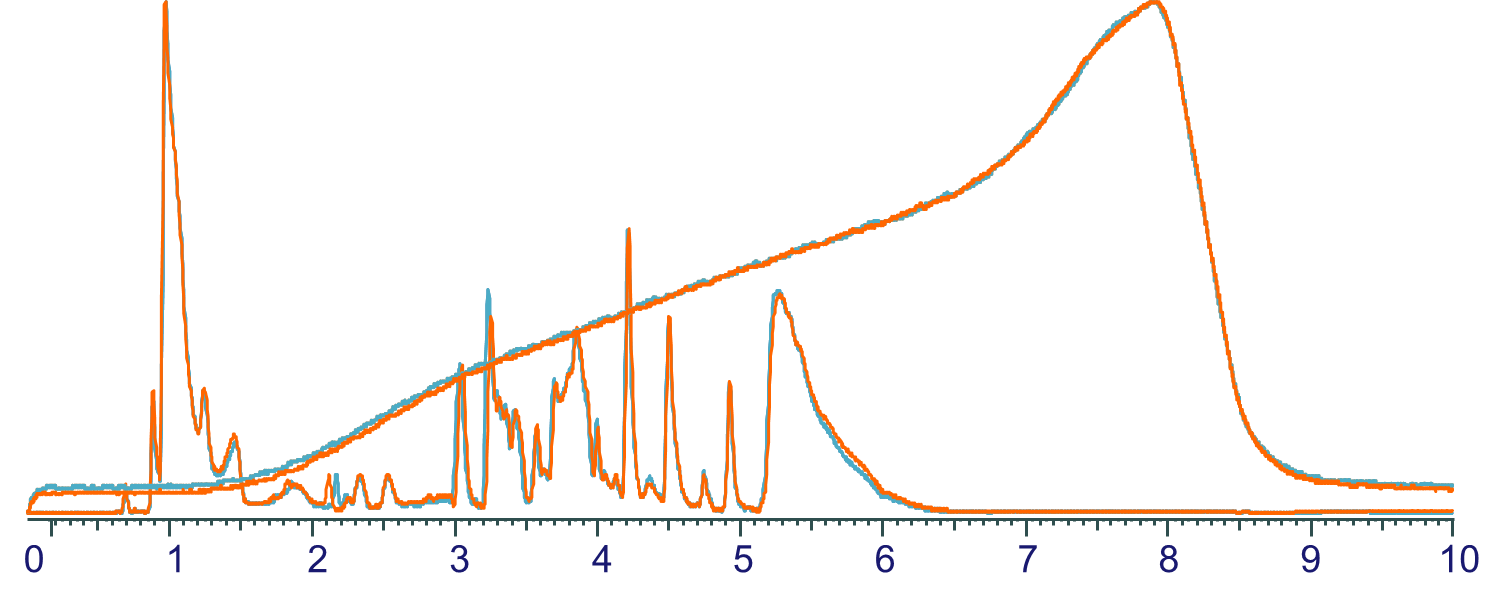


**Figure S6:** Overlapped traces of base peak chromatogram and relative backpressure in HILIC-HRMS from #1 to 250 consecutive injection


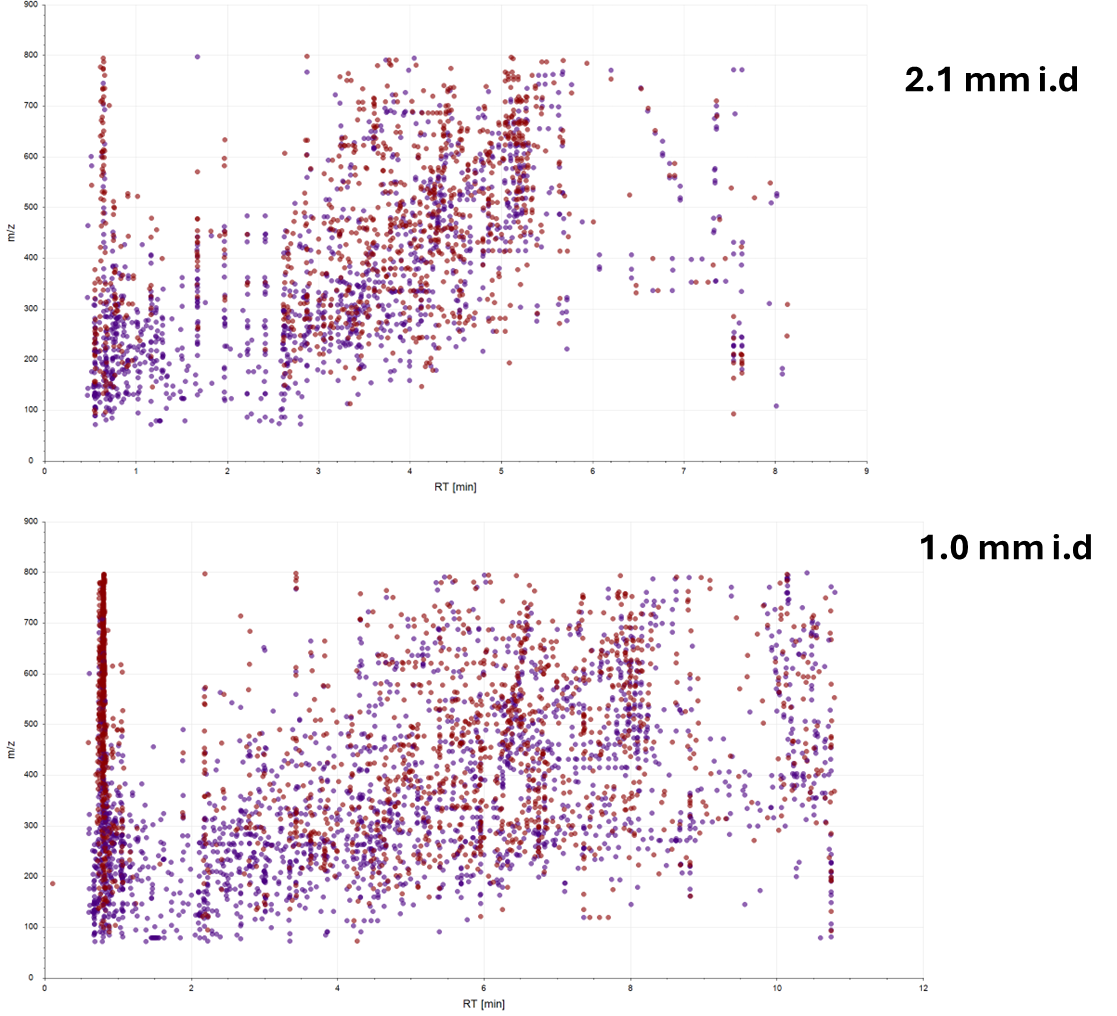


**Figure S7:** Dot plots of MS1 and MS/MS events on 2.1 mm i.d (top) and 1.0 mm i.d (bottom) setups


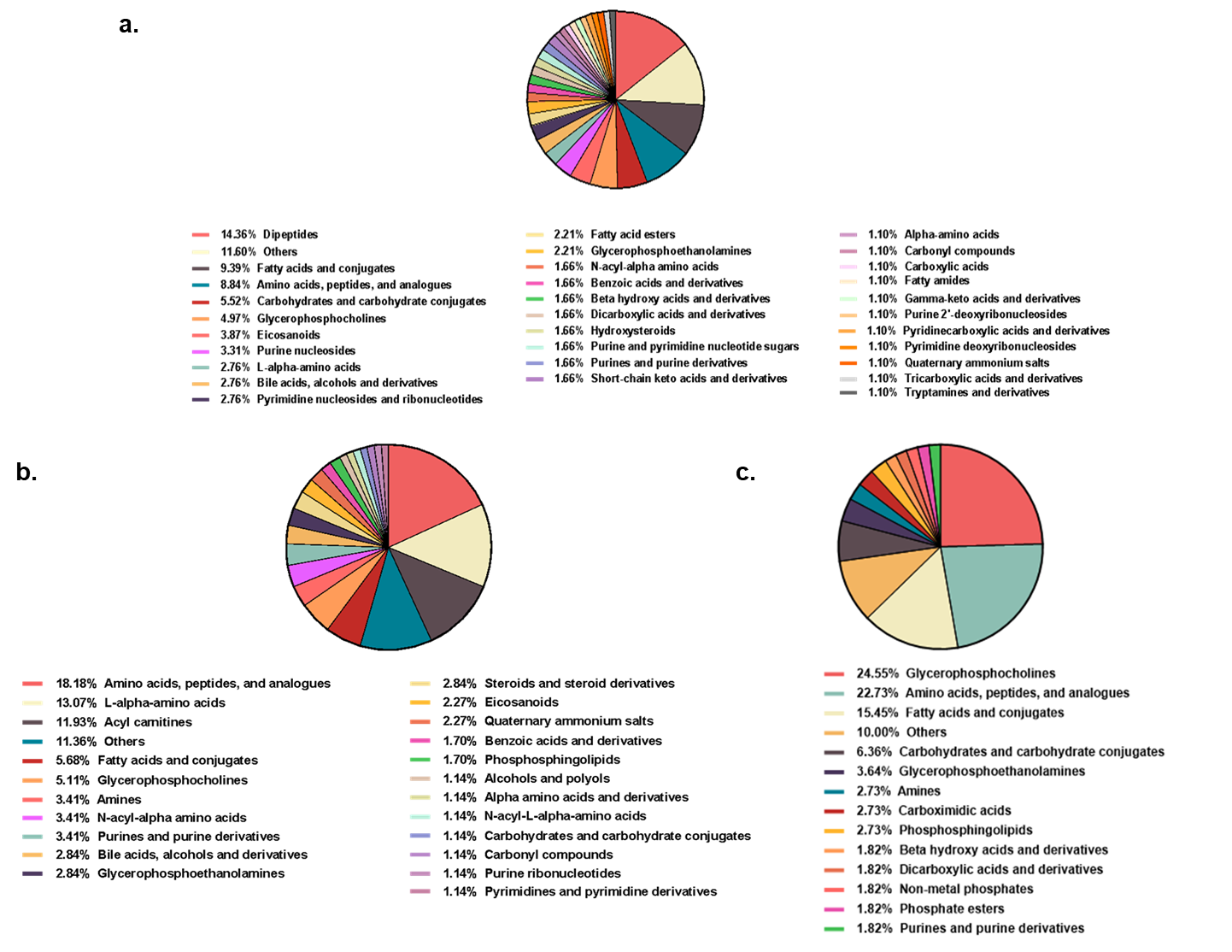


**Figure S8a-c:** Graphical representation of metabolome coverage and overlap between 1.0 and 2.1 mm i.d
